# Association between the imbalance in peripheral lymphocyte subsets and pneumonia-associated acute respiratory distress syndrome in adults: a cross-sectional study

**DOI:** 10.3389/fmed.2026.1753313

**Published:** 2026-04-01

**Authors:** Zhen-Chuan Xing, Hua-Zheng Guo, Ting Ao, Jin-Xiang Wang, Ming Hu

**Affiliations:** 1 Department of Pulmonary and Critical Care Medicine, Beijing Luhe Hospital, Capital Medical University, Beijing, China; 2 Department of Infectious Disease, Beijing Luhe Hospital, Capital Medical University, Beijing, China

**Keywords:** acute respiratory distress syndrome, immunophenotyping, lymphocyte subsets, pneumonia, risk factors

## Abstract

**Background:**

Acute respiratory distress syndrome (ARDS) is a severe complication of pneumonia that significantly increases mortality. Its pathogenesis is associated with dysregulated host immune responses, which has been a topic of global research in lung injury.

**Methods:**

In this cross-sectional study, we enrolled adult patients with pneumonia from the Department of Infectious Diseases between May 2021 and June 2025. Participants were categorized into ARDS and non-ARDS groups based on the presence of ARDS at admission. We employed multivariable logistic regression and restricted cubic spline models to evaluate the association between lymphocyte subset levels and ARDS, adjusting for key clinical confounders.

**Results:**

Of the 227 eligible patients, 127 were diagnosed with ARDS. Patients with ARDS exhibited lower CD3^+^CD4^+^ T-cell counts compared to non-ARDS controls. Furthermore, CD3^+^CD4^+^ T-cell counts decreased progressively with increasing ARDS severity (*P* for trend <0.001). After comprehensive adjustment, a twofold higher CD3^+^CD4^+^ T-cell count was significantly associated with 33% lower odds of presenting with ARDS (adjusted OR 0.67, 95% CI 0.48–0.95), with evidence of a dose–response relationship. A nonlinear threshold effect was observed, with a significant inverse association noted above 227 cells/μL. This relationship remained consistent across all prespecified subgroups, with no evidence of effect modification.

**Conclusion:**

This study indicates an independent inverse association between CD3^+^CD4^+^ T-cell counts and the presence of pneumonia-related ARDS. However, the cross-sectional design precludes any conclusions about its clinical applicability. Further research in prospective cohorts is warranted.

## 1 Introduction

Pneumonia is a major global clinical and public health issue. It is remain a leading cause of mortality worldwide, resulting in an estimated 2.4 million deaths annually according to a recent World Health Organization report (1). Acute Respiratory Distress Syndrome (ARDS) is a devastating pulmonary complication associated with uncontrolled systemic inflammation and diffuse alveolar damage (2). The mortality risk in patients with pneumonia-associated ARDS is significantly higher than in those with pneumonia alone (3). The pathogenesis of ARDS is complex, with dysregulated host immune response (4). The initial insult, such as a pulmonary infection, triggers an exuberant inflammatory cascade, leading to the release of a flood of cytokines and chemokines, and the recruitment of neutrophils into the alveolar space (5). This process results in damage to the alveolar-capillary membrane and impaired gas exchange. Crucially, this pro-inflammatory state is often accompanied by a concurrent state of immune suppression, a phenomenon frequently observed in severe sepsis and ARDS (6). The balance between pro-inflammation and anti-inflammation, as well as between innate and adaptive immunity, is critical in determining patient outcomes (7).

Lymphopenia has been consistently recognized as a hallmark of severe infection and is a strong predictor of mortality in septic patients (8). However, the total lymphocyte count is a crude measure that fails to capture the complexity of immune dysregulation. The analysis of lymphocyte subsets offers a more precise and nuanced reflection of the immune status than total lymphocyte count alone, as changes in specific populations can be quantified (9). While the clinical application of lymphocyte subset analysis is expanding in fields like oncology and transplantation, its associations with ARDS in the context of pneumonia remain inadequately explored.

In recent years, the role of the immune system in lung injury has attracted extensive attention worldwide (10). Emerging evidence suggests that low CD4^+^ T-cell counts are associated with adverse outcomes in sepsis and COVID-19-related ARDS (11, 12), yet comprehensive data in general pneumonia populations are lacking. Therefore, to address this gap, our study aims to preliminarily investigate the correlation between peripheral blood lymphocyte subset levels and the presence of ARDS in patients with pneumonia. These findings may provide valuable insights for understanding the immune profile associated with ARDS and inform the design of future prospective studies.

## 2 Materials and methods

### 2.1 Study population

**Figure 1:**
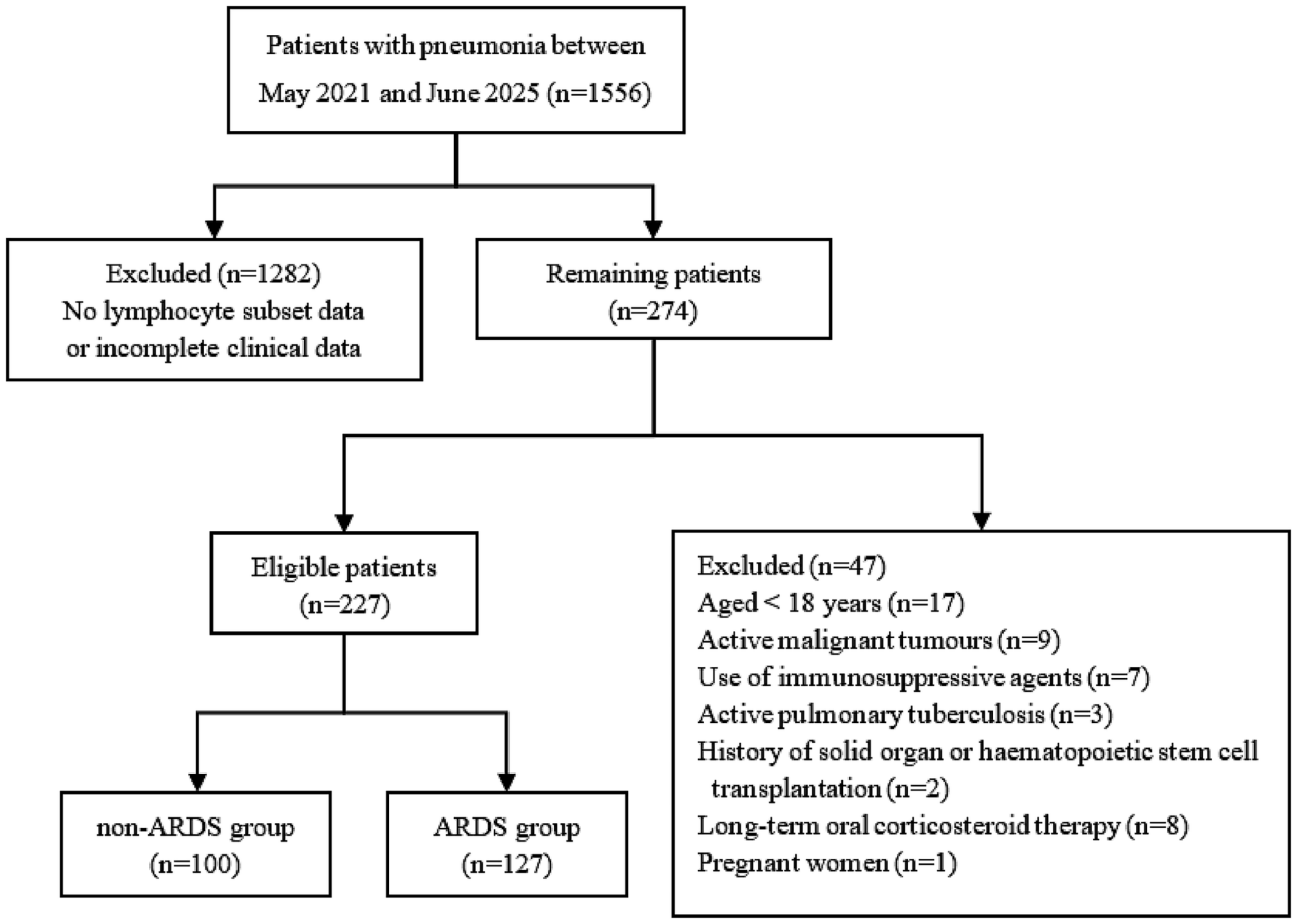
Flow chart of inclusion and exclusion criteria of participants.

This study was a single-center retrospective study. A total of 227 adult patients with pneumonia admitted to the Department of Infectious Diseases, Beijing Luhe Hospital, Capital Medical University, between May 2021 and June 2025 were enrolled. Inclusion criteria: (i) age ≥ 18 years; (ii) diagnosis of pneumonia meets the criteria established by the ATS/IDSA guideline (13); and (iii) peripheral blood lymphocyte subset analysis was performed within 48 h of admission. Exclusion criteria: (i) active malignancy; (ii) use of immunosuppressive agents; (iii) active pulmonary tuberculosis; (iv) history of solid organ or hematopoietic stem cell transplantation; (v) long-term oral corticosteroid therapy; (vi) pregnancy; and (vii) significant limitations in clinical assessment due to incomplete medical data. Based on the presence of ARDS at admission, patients were stratified into two cohorts: a non-ARDS group (*n* = 100) and an ARDS group (*n* = 127). This study aimed to compare intergroup clinical and laboratory variables, with a focus on lymphocyte subset characteristics, and to evaluate their cross-sectional association with ARDS (Figure 1). The study was approved by the ethics committee of Beijing Luhe Hospital (2025-LHKY-017-01). Patients information was collected through the Hospital Information System (HIS).

### 2.2 Definitions

#### 2.2.1 Definitions of pneumonia

Pneumonia was diagnosed based on the criteria established by the 2019 American Thoracic Society/Infectious Diseases Society of America (ATS/IDSA) clinical practice guideline (13). The diagnosis required the presence of both: (1) Clinical Features: At least one of the following clinical criteria: acute onset of cough (with or without sputum production), fever, pleuritic chest pain, or signs of systemic inflammation (e.g., leukocytosis or leukopenia); and (2) Radiological Evidence: A new or progressive pulmonary infiltrate on chest radiography or computed tomography (CT) scan, consistent with pneumonia.

#### 2.2.2 Definitions of ARDS

The diagnosis of ARDS was established according to the Berlin Definition (14), which requires the simultaneous presence of all the following criteria: (1) onset within 1 week of a known clinical insult or new/worsening respiratory symptoms; (2) bilateral opacities on chest imaging not fully explained by effusions, lobar/lung collapse, or nodules; (3) respiratory failure not fully explained by cardiac failure or fluid overload; and (4) impaired oxygenation, as defined by the PaO₂/FiO₂ ratio ≤300 mmHg measured with a positive end-expiratory pressure of at least 5 cm H₂O. Patients diagnosed with ARDS were categorized into three groups: mild ARDS (200 mmHg <PaO₂/FiO₂ ≤ 300 mmHg), moderate ARDS (100 mmHg <PaO₂/FiO₂ ≤ 200 mmHg), and severe ARDS (PaO₂/FiO₂ ≤ 100 mmHg).

#### 2.2.3 CURB-65 score

The CURB-65 score was calculated at admission based on five components, each assigned one point: (1) Confusion (new-onset disorientation); (2) Urea >7 mmol/L; (3) Respiratory rate ≥30 breaths/min; (4) Hypotension defined as systolic blood pressure <90 mmHg or diastolic blood pressure ≤60 mmHg; and (5) Age ≥65 years.

### 2.3 Data collection

The demographic and clinical data collected including: (1) age and gender; (2) comorbidities (chronic pulmonary disease, diabetes, cerebrovascular disease, ischemic heart disease, hypertension); (3) vital signs measured at admission, including respiratory rate, blood pressure, heart rate, and altered mental status; (4) peripheral lymphocyte subset; (5) laboratory measures: white blood cell count (WBC), neutrophil count, lymohocyte count, platelet count (PLT), procalcitonin (PCT), C-reactive protein (CRP), interleukin-6 (IL-6), serum ferritin, alanine aminotransferase (ALT), aspartate aminotransferase (AST), albumin (ALB), total bilirubin (Tbil), serum creatinine, urea, creatine kinase (CK), lactate dehydrogenase (LDH), prothrombin time (PT), activated partial thromboplastin time (APTT), thrombin time (TT), fibrinogen, D-Dimer, and PaO₂/FiO₂ ratio; (6) pathogen, clinical outcome; and (7) CURB-65 score components and total score. Lymphocyte subset analyses and laboratory assessments were performed within 48 h after admission.

### 2.4 Flow cytometric analysis of lymphocyte subpopulations

Peripheral blood samples were collected from participants via venipuncture into K₂EDTA or K₃EDTA vacuum tubes (BD Vacutainer). Within 24 h of collection, 50 μL of thoroughly mixed whole blood was aliquoted into a pre-labeled BD Trucount™ absolute counting tube. Subsequently, 20 μL of the BD Multitest 6-Color TBNK reagent (BD Biosciences, Cat. No. 662967) was added directly to the blood. The tube was vortexed gently and incubated for 15 min in the dark at room temperature to allow for antibody binding. Following incubation, red blood cells were lysed by adding 450 μL of a pre-diluted (1X) BD FACS™ Lysing Solution (BD Biosciences) and incubating for another 15 min in the dark. The samples were then acquired on a BD FACSLyric™ flow cytometer (or BD FACSCanto™ II), which was calibrated daily using BD CS&T Beads and BD 7-Color Setup Beads to ensure optimal performance and fluorescence compensation. A minimum of 70,000 events within the lymphocyte gate, as identified by CD45 expression and side scatter characteristics, were acquired for each sample. The absolute counts (expressed as cells/μL) for each lymphocyte subset were automatically calculated by the BD FACSuite™ software using the single-platform Trucount methodology, which relies on the known ratio of acquired cellular events to the internal fluorescent bead standard. The following lymphocyte subpopulations were identified and quantified using this standardized 6-color panel: Total T lymphocytes (CD3^+^), Helper T cells (CD3^+^CD4^+^), Cytotoxic T cells (CD3^+^CD8^+^), B lymphocytes (CD19^+^), Natural Killer (NK) cells CD3^−^ (CD16^+^ and/or CD56^+^).

### 2.5 Statistical analysis

The characteristics of all individuals were summarized based on the presence or absence of ARDS. Normally distributed continuous variables were presented as mean ± standard deviation (SD) and were compared between groups using the independent samples *t*-test. Non-normally distributed continuous variables were expressed as median and interquartile range (IQR) and were compared using the Mann–Whitney U test. Categorical variables were reported as frequencies and percentages, and group differences were assessed using the chi-square test or Fisher’s exact test. We employed five-fold cross-validation in conjunction with least absolute shrinkage and selection operator (LASSO) regression to analyze variables with *p*-values <0.1 from the initial univariate analysis. Subsequently, logistic regression was applied to estimate the odds ratios (OR) and 95 percent confidence intervals (95% CIs) for the association between lymphocyte subsets and ARDS. Six progressively adjusted models were used. Model1 was severed as an unadjusted or crude model. Model 2 was adjusted for age and sex. Model 3 was further adjusted for albumin, Tbil, neutrophil count, C-reactive protein (CRP), and procalcitonin (PCT) in addition to the variables in model 2. Model 4 included all variables from model 3 and additionally accounted for comorbidities, including cerebrovascular disease and heart failure. To address potential confounding by disease severity, we performed a sensitivity analysis by further adjusting for the CURB-65 score. This model, designated as Model 5, included all covariates from Model 4 plus the CURB-65 score. Additionally, to assess whether the inclusion of inflammatory markers (CRP, PCT, and neutrophil count) might introduce over-adjustment bias, we constructed Model 6 by removing these three variables from Model 4 while retaining all other covariates, to reassess the association between CD4^+^ T cell counts and ARDS.

In the restricted cubic spline (RCS) analysis, we employed a binary logistic regression model to evaluate the nonlinear relationship between CD4^+^ T cell count and ARDS. The model included ARDS as the dependent variable and CD4^+^ T cell count as the primary independent variable, with adjustment for age, sex, and covariates identified by LASSO regression (albumin, total bilirubin, neutrophil count, C-reactive protein, procalcitonin, cerebrovascular disease, and heart failure). Odds ratios (OR) and 95% confidence intervals (CI) were calculated to estimate the effect size. To flexibly fit potential nonlinear relationships, four knots were placed at automatically selected percentiles based on the distribution of CD4^+^ T cell counts. A nonlinearity test was conducted to assess the statistical significance of deviation from linearity. To further assess the robustness of the inflection point and to construct its confidence interval, we applied a bootstrap resampling method as an internal validation strategy. Specifically, 1,000 bootstrap samples were drawn with replacement from the original dataset. In each bootstrap sample, the RCS-based logistic regression model was refitted, and the inflection point was estimated. The distribution of these 1,000 bootstrap-derived inflection points was then used to obtain the median inflection point and its 95% percentile-based confidence interval.

Subgroup analyses were performed to evaluate the consistency of the relationship between lymphocyte subset and ARDS across various populations. These analyses were stratified by sex (male vs. female), age (<65 vs. ≥65 year), cerebrovascular disease (yes vs. no), heart failure (yes vs. no), COVID-19 (yes vs. no), and hypotension at admission (yes vs. no), defined as an initial systolic blood pressure (SBP) < 90 mmHg or a mean arterial pressure (MAP) < 65 mmHg. A two-sided *p*-value <0.05 was considered statistically significant. Statistical analyses were performed using Python Software (3.7.1, http://www.python.org) and the Free Statistics analysis platform (Version 2.4, Beijing, China).

## 3 Results

### 3.1 Clinical characteristics of patients with pneumonia

This study comprised 227 patients with pneumonia, with a mean age of 72.8 ± 11.6 years (range, 39–98) and a male predominance of 66.1%. Hypertension was the most prevalent comorbidity (136, 59.9%), followed by diabetes mellitus (84, 37.0%), heart failure (83, 36.6%), and cerebrovascular disease (74, 32.6%). COVID-19 was the most frequently identified pathogen (81, 35.7%). Among them, 127 (55.9%) were diagnosed with ARDS at admission. The baseline characteristics between the non-ARDS and ARDS groups were largely comparable in terms of sex, age, and most comorbidities. However, patients in the ARDS group had a significantly higher prevalence of heart failure (55.9% vs. 12.0%, *p* < 0.001) and cerebrovascular disease (40.9% vs. 22.0%, *p* = 0.003), but a lower prevalence of ischemic heart disease (18.1% vs. 30.0%, *p* = 0.037). Notable differences were observed in admission laboratory profiles. Compared to patients without ARDS, those diagnosed with ARDS exhibited a more pronounced inflammatory response and greater evidence of organ injury upon hospital admission. The ARDS group demonstrated substantially elevated levels of white blood cell count, neutrophil count, C-reactive protein, procalcitonin, and interleukin-6 (all *p* < 0.05). Conversely, lymphocyte counts and albumin levels were significantly lower in ARDS patients (*p* < 0.05). Furthermore, markers of coagulation dysfunction and organ damage were also significantly higher in the ARDS cohort. This included D-dimer, total bilirubin, Serum creatinine, urea, and lactate dehydrogenase (all *p* < 0.05). No significant differences were detected in the distribution of viral or fungal pathogens between the groups. The ARDS cohort exhibited both a significantly longer median hospital length of stay (14.0 vs. 8.0 days, *p* < 0.001) and a higher in-hospital mortality rate (35.4% vs. 4.0%, *p* < 0.001) (Table 1).

**Table 1:** Baseline clinical characteristics of patients with pneumonia.

Variables	Total (*n* = 227)	Non-ARDS (*n* = 100)	ARDS (*n* = 127)	*P*
Male, *n* (%)	150 (66.1)	61 (61.0)	89 (70.1)	0.152
Age, (years)	72.8 ± 11.6	71.3 ± 12.8	73.9 ± 10.4	0.093
Comorbidities				
Hypertension, *n* (%)	136 (59.9)	56 (56.0)	80 (63.0)	0.286
Ischemic heart disease, *n* (%)	53 (23.3)	30 (30.0)	23 (18.1)	0.037
Cerebrovascular disease, *n* (%)	74 (32.6)	22 (22.0)	52 (40.9)	0.003
Diabetes mellitus, *n* (%)	84 (37.0)	31 (31.0)	53 (41.7)	0.097
Chronic pulmonary disease, *n* (%)	36 (15.9)	16 (10.0)	20 (15.7)	0.959
Chronic liver disease, *n* (%)	4 (1.8)	3 (3.0)	1 (0.8)	0.242
Chronic kidney disease, *n* (%)	37 (16.3)	14 (14.0)	23 (18.1)	0.406
Heart failure, *n* (%)	83 (36.6)	12 (12.0)	71 (55.9)	<0.001
Laboratory test at admission				
White blood cell, (×10^9^/L)	8.4 ± 5.3	6.8 ± 3.8	9.7 ± 6.0	<0.001
Neutrophil, (×10^9^/L)	6.2 (3.5, 9.7)	4.5 (3.0, 7.7)	7.3 (4.2, 11.0)	<0.001
Lymohocyte, (×10^9^/L)	0.8 ± 0.5	0.9 ± 0.5	0.7 ± 0.4	<0.001
Platelet, (×10^9^/L)	187.9 ± 87.0	186.4 ± 79.3	189.0 ± 92.9	0.822
C-reactive protein, (mg/L)	95.9 (43.8, 194.8)	64.7 (30.5, 117.9)	142.3 (62.1, 200.0)	<0.001
Procalcitonin, (ng/mL)	0.2 (0.1, 1.6)	0.1 (0.1, 0.3)	0.5 (0.1, 6.2)	0.004
Interleukin-6, (pg/mL)	50.2 (13.2, 201.4)	32.2 (9.1, 99.4)	79.5 (24.7, 450.0)	0.026
Serum ferritin,(ng/mL)	615.9 (299.9, 1107.9)	483.7 (256.5, 962.0)	675.9 (342.7, 1257.4)	0.075
Prothrombin time, (S)	13.0 ± 3.6	12.7 ± 4.1	13.2 ± 3.1	0.286
APTT, (S)	32.0 ± 6.7	32.2 ± 7.2	31.8 ± 6.4	0.695
Thrombin time, (S)	14.0 (13.2, 15.4)	13.9 (13.2, 15.2)	14.1 (13.2, 15.6)	0.717
Fibrinogen, (g/L)	5.1 ± 2.0	5.2 ± 1.5	5.2 ± 2.3	0.609
D-Dimer, (μg/mL)	0.6 (0.3, 1.3)	0.4 (0.2, 0.8)	0.9 (0.5, 2.2)	0.003
Albumin, (g/L)	33.5 ± 4.8	35.2 ± 4.8	32.1 ± 4.5	<0.001
ALT, (U/L)	20.0 (13.0, 36.5)	20.0 (14.0, 34.2)	21.0 (12.5, 41.0)	0.439
AST, (U/L)	27.0 (18.0, 41.0)	25.0 (18.0, 33.0)	29.0 (18.5, 46.5)	0.245
Total bilirubin, (μmol/L)	10.7 ± 7.1	9.2 ± 5.2	11.8 ± 8.1	0.007
Serum creatinine, (μmol/L)	80.0 (63.5, 113.5)	79.0 (64.0, 100.2)	83.0 (63.0, 155.5)	0.021
Urea, (mmol/L)	7.7 (5.1, 12.7)	6.7 (4.7, 8.9)	9.2 (6.0, 15.0)	<0.001
Creatine kinase, (U/L)	85.0 (42.0, 222.5)	67.5 (35.5, 156.0)	101.0 (49.0, 265.0)	0.133
Lactate dehydrogenase, (U/L)	240.0 (181.0, 323.0)	218.5 (173.8, 256.0)	276.0 (200.0, 378.5)	0.015
Pathogen				
COVID-19, *n* (%)	81 (35.7)	39 (39.0)	42 (33.1)	0.355
Aspergillus, *n* (%)	10 (4.4)	2 (2.0)	8 (6.3)	0.137
Influenza, *n* (%)	11 (4.8)	4 (4.0)	7 (5.5)	0.600
Hospital length of stay, (days)	10.0 (7.0, 16.5)	8.0 (6.0, 11.0)	14.0 (8.5, 20.5)	<0.001
In-hospital mortality, *n* (%)	49 (21.6)	4 (4.0)	45 (35.4)	<0.001

The *P*-values denote the statistical significance of comparisons between the non-ARDS and ARDS groups. Alanine aminotransferase (ALT), Aspartate aminotransferase (AST), Activated partial thromboplastin time (APTT).

### 3.2 Lymphocyte subsets

The absolute count of total lymphocytes, CD3^+^ T-cell, CD3^+^CD8^+^ T-cell, CD3^+^CD4^+^ T-cell, and CD16^+^CD56^+^ NK cell in the ARDS group were significantly lower than those in the non-ARDS group (all *p* < 0.001). There were no significant difference in CD19^+^ B-cell and CD4+/CD8 + ratio between the two groups (*p* > 0.05) (Table 2).

**Table 2:** Comparisons of lymphocyte subsets between the ARDS and non-ARDS patients.

Variables	Normal ranges /μL	Total (*n* = 227)	Non-ARDS (*n* = 100)	ARDS (*n* = 127)	*P*
Total lymphocytes /μL	800–4,000	887.0 ± 520.3	1040.3 ± 515.1	766.2 ± 493.7	<0.001
T cells (CD3+) /μL	940–2,140	485.0 (267.5, 756.0)	677.0 (340.2, 898.5)	363.0 (239.5, 617.5)	<0.001
T cells (CD3 + CD8+) /μL	380–790	160.0 (94.0, 270.0)	211.0 (107.0, 294.8)	127.0 (75.5, 251.0)	<0.001
T cells (CD3 + CD4+) /μL	550–1,200	235.0 (143.0, 426.0)	378.0 (204.8, 533.8)	193.0 (121.5, 332.0)	<0.001
NK cells (CD16 + CD56+) /μL	155–550	131.0 (71.0, 229.0)	153.5 (104.2, 250.5)	107.0 (54.0, 213.0)	<0.001
B cells (CD19+) /μL	160–350	97.0 (55.5, 166.5)	108.0 (61.8, 193.5)	85.0 (47.0, 150.5)	0.052
CD4+/CD8 + ratio	0.9–2.0	1.6 (1.0, 2.4)	1.6 (1.2, 2.2)	1.5 (0.9, 2.4)	0.597

NK cell, natural killer cells. The *P*-values denote the statistical significance of comparisons between the non-ARDS and ARDS groups.

### 3.3 Association between lymphocyte subsets and ARDS

**Figure 2:**
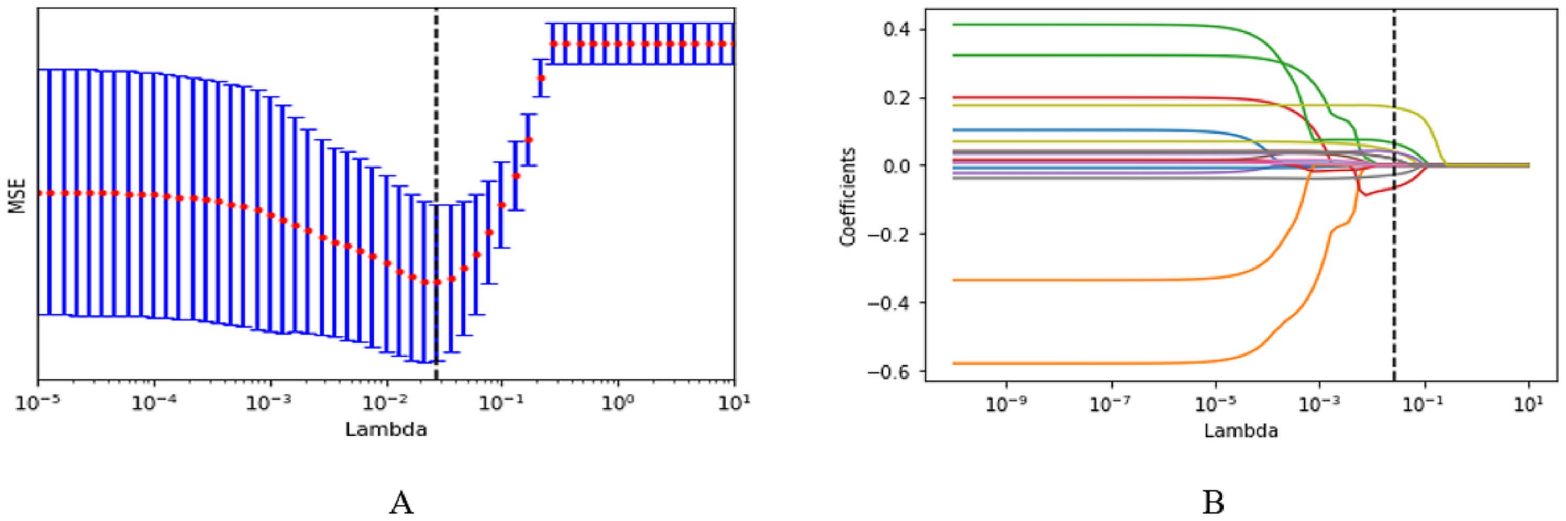
Variable selection via least absolute shrinkage and selection operator (LASSO) regression. **(A)** Identification of the optimal penalty parameter (*λ*) through 5-fold cross-validation; **(B)** Final variables selected at the optimal lambda value.

We applied a Least Absolute Shrinkage and Selection Operator (LASSO) regression model with five-fold cross-validation for identifying variables associated with ARDS, using its presence as the dependent variable and the 23 candidate variables (with *p* < 0.1 from univariate analyses in Tables 1, 2) as independents. This approach ultimately retained 8 non-zero coefficients, including the CD3^+^CD4^+^ T cells count, albumin (ALB), total bilirubin (Tbil), neutrophil count, C-reactive protein (CRP), procalcitonin (PCT), cerebrovascular disease, and heart failure (Figure 2).

In logistic regression analysis, T cells (CD3 + CD4+) counts were log2-transformed to better capture their nonlinear relationship with ARDS. The eight variables selected by LASSO, as well as age and sex, were subsequently included in separate logistic regression analyses. In the initial model, a significant inverse association was observed between Log2 (T cells CD3 + CD4+) and the odds of ARDS (OR 0.57; 95% CI, 0.43–0.74; *p* < 0.001). Specifically, an association was observed between each twofold increase in CD3^+^CD4^+^ T-cell count and 43% lower odds of ARDS. This inverse association remained consistent across sequentially adjusted models: after adjustment for age and sex (Model 2: OR 0.58; 95% CI, 0.44–0.76; *p* < 0.001), further adjustment for laboratory markers including albumin, total bilirubin, neutrophil count, C-reactive protein, and procalcitonin (Model 3: OR 0.65; 95% CI, 0.48–0.90; *p* = 0.009), after additional adjustment for cerebrovascular disease and heart failure (Model 4: OR 0.67; 95% CI, 0.48–0.95; *p* = 0.023), and after further adjustment for CURB-65 score (Model 5: OR 0.70; 95% CI, 0.49–1.00; *p* = 0.05). In an additional model adjusted for age, sex, albumin, total bilirubin, cerebrovascular disease, and heart failure, while excluding neutrophil count, C-reactive protein, and procalcitonin to assess the influence of inflammatory markers (Model 6), the association remained statistically significant (OR 0.66; 95% CI, 0.48–0.90; *p* = 0.008).

To further assess the impact of CD3 + CD4 + T-cell count, patients were divided into three equal groups based on their values: T1, T2, and T3. The analysis across these strata confirmed that individuals in the highest CD3 + CD4 + T-cell count group had significantly decreased odds of presenting with ARDS compared to those in the lowest group in the fully adjusted model (Model 5: adjusted OR 0.32; 95% CI, 0.13–0.83; *p* = 0.019). In addition, a trend test indicated a statistically significant trend (*p* = 0.012) toward lower odds with increasing CD3 + CD4 + T-cell count levels (Table 3).

**Table 3:** Association between T cells (CD3 + CD4+) count and ARDS in different models.

Variables	Model 1		Model 2		Model 3		Model 4		Model 5		Model 6	
	OR(95%CI)	*P*	OR(95%CI)	*P*	OR(95%CI)	*P*	OR(95%CI)	*P*	OR(95%CI)	*P*	OR(95%CI)	*P*
Log2 T cells CD3 + CD4+	0.57 (0.43 ~ 0.74)	<0.001	0.58 (0.44 ~ 0.76)	<0.001	0.65 (0.48 ~ 0.90)	0.009	0.67 (0.48 ~ 0.95)	0.023	0.70 (0.49 ~ 1.00)	0.050	0.66 (0.48 ~ 0.90)	0.008
T cells CD3 + CD4+. Tertiles												
T1	1 (Ref)		1 (Ref)		1 (Ref)		1 (Ref)		1 (Ref)		1 (Ref)	
T2	0.87 (0.44 ~ 1.72)	0.685	0.87 (0.44 ~ 1.74)	0.694	1.15 (0.53 ~ 2.53)	0.721	1.60 (0.67 ~ 3.81)	0.288	1.78 (0.71 ~ 4.46)	0.221	1.57 (0.70 ~ 3.53)	0.273
T3	0.20 (0.10 ~ 0.40)	<0.001	0.21 (0.10 ~ 0.42)	<0.001	0.26 (0.12 ~ 0.60)	0.002	0.31 (0.13 ~ 0.75)	0.010	0.32 (0.13 ~ 0.83)	0.019	0.27 (0.12 ~ 0.61)	0.002
Trend test		<0.001		<0.001		<0.001		0.005		0.012		0.001

Log2 (T cells CD3 + CD4+): CD3 + CD4 + T-cell counts were log2-transformed for analysis. The odds ratio (OR) corresponds to a twofold increase in the original cell count; OR, odds ratio; CI, confidence interval.

Model 1: unadjusted.

Model 2: adjusted for sex, age.

Model 3: adjusted for model 2 + albumin, Tbil, neutrophil count, C-reactive protein (CRP), procalcitonin (PCT).

Model 4: adjusted for model 3 + cerebrovascular disease, heart failure.

Model 5: adjusted for model 4 + curb-65 score.

Model 6:adjusted for model 2 + albumin, Tbil, cerebrovascular disease, heart failure.

**Figure 3:**
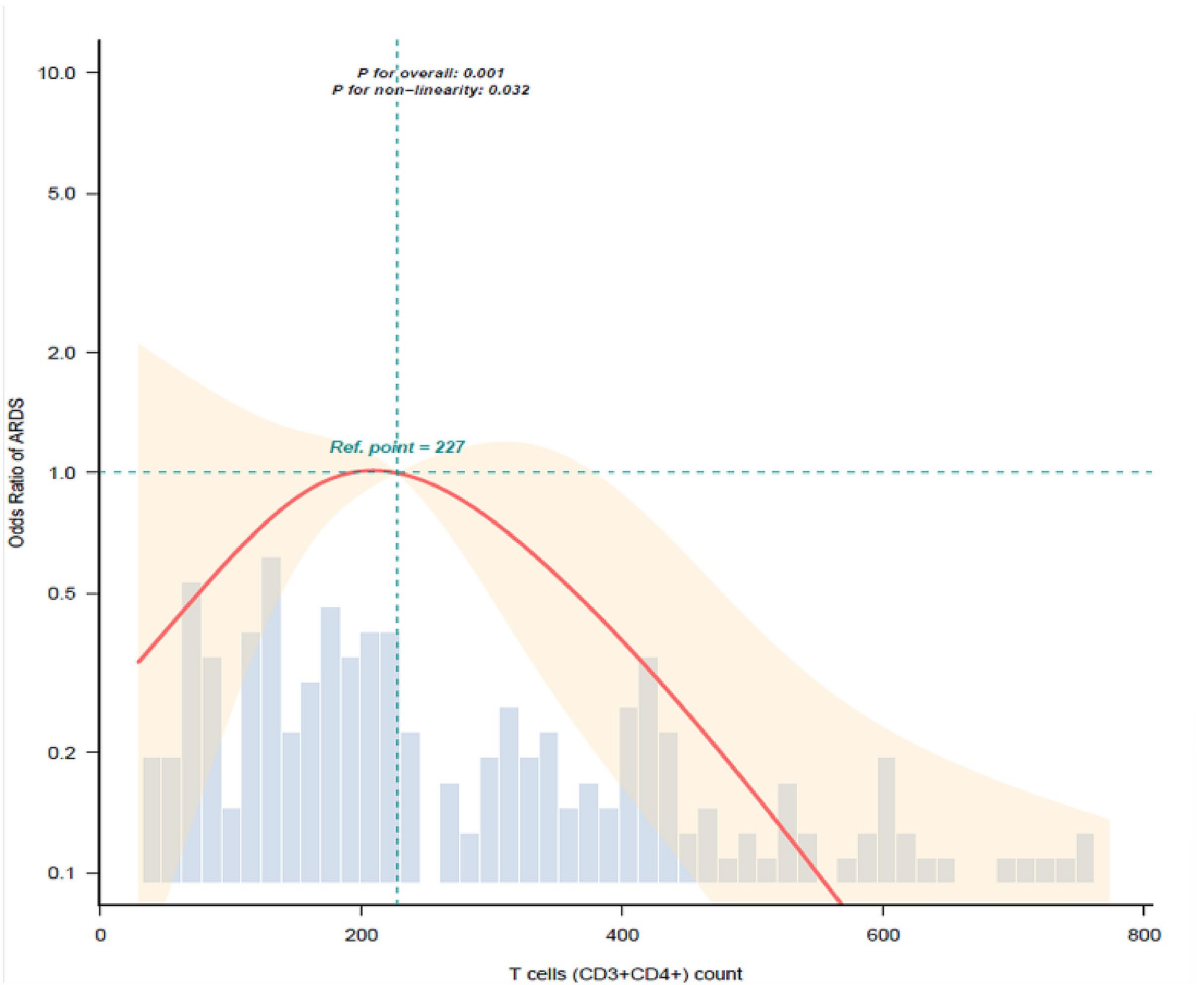
Restricted cubic spline curve describing the dose–response relationship between T cells CD3^+^CD4^+^ count and pneumonia-associated ARDS. Adjusted for age, sex, albumin, Tbil, neutrophil count, C-reactive protein (CRP), procalcitonin (PCT), cerebrovascular disease, and heart failure. Only 95% of the data is displayed. OR, odds ratio; CI, confidence interval. The solid red line represents the estimated OR, and the shaded area represents the 95% confidence interval. The reference value (OR = 1) was set at 227 cells/μL.

The relationship between T cells (CD3 + CD4+) count and the odds of ARDS was examined. To elucidate this relationship independent of potential confounders, a multivariable model was constructed using age, sex, and the variables selected by LASSO regression. When analyzed as a continuous variable, CD3 + CD4 + T-cell count was significantly associated with the likelihood of ARDS. Following adjustment for potential confounders, each 50-cell/μL increment in CD3 + CD4 + T-cell count corresponded to an 11% reduction in the likelihood of ARDS (OR = 0.89, 95% CI: 0.82–0.97, *p* = 0.009). Similarly, each 100-cell/μL increase was associated with a 20% lower likelihood of ARDS (OR = 0.80, 95% CI: 0.67–0.94, *p* = 0.009). Exploratory threshold effect analysis using restricted cubic splines (RCS) revealed a significant non-linear relationship between CD4^+^ T-cell count and ARDS (*P* for non-linearity = 0.029), with a potential inflection point identified at 227 cells/μL. Below this threshold (<227 cells/μL), each 50-cell/μL increment in CD4^+^ T-cell count was significantly associated with a lower likelihood of ARDS, with an adjusted odds ratio (OR) of 0.832 (95% CI, 0.735–0.941, *p* = 0.0035). Conversely, above the threshold (≥227cells/μL), each 50-cell/μL increment was associated with a non-significantly higher likelihood of ARDS (OR = 1.244, 95% CI: 0.646–2.396, *p* = 0.5130) (Figure 3; Table 4). To evaluate the stability of the threshold value at 227 cells/μL, we performed bootstrap resampling analysis. We generated 1,000 bootstrap samples from the original dataset (*n* = 227) using sampling with replacement. In each bootstrap sample, we refitted a multivariable-adjusted restricted cubic spline model. Among the 1,000 replicates, the median threshold value was 235.3 cells/μL, with a 95% bootstrap confidence interval of 210.7–238.8 cells/μL.

**Table 4:** Association between CD3 + CD4^+^ T-cell count and ARDS: dose–response relationship and threshold effect analysis.

Variables	Adjusted OR (95% CI)	*p*-value
T cells CD3 + CD4 + count (continuous variable)		
Per 1-cell/μL increase	1.00 (1.00 ~ 1.00)	0.009
Per 50-cell/μL increase	0.89 (0.82 ~ 0.97)	0.009
Per 100-cell/μL increase	0.80 (0.67 ~ 0.94)	0.009
Threshold effect analysis		
≥227 cells/μL (per 50-cell/μL increase)	1.244 (0.646 ~ 2.396)	0.5130
<227 cells/μL (per 50-cell/μL increase)	0.832 (0.735 ~ 0.941)	0.0035

Analysis adjusted for age, sex, albumin, Tbil, neutrophil count, C-reactive protein (CRP), procalcitonin (PCT), cerebrovascular disease, and heart failure. OR, odds ratio; CI, confidence interval.

Based on the Berlin definition of ARDS severity, we further analyzed the distribution of CD3^+^CD4^+^ T-cell counts. Among the 227 enrolled patients, 100 were classified as non-ARDS, and 127 met the diagnostic criteria for ARDS, including 55 (43.3%) with mild, 54 (42.5%) with moderate, and 18 (14.2%) with severe ARDS. As shown in Table 5, CD3^+^CD4^+^ T-cell counts decreased progressively with increasing ARDS severity. The median CD3 + CD4^+^ T-cell count was 378.0 (IQR: 204.8–533.8) cells/μL in the non-ARDS group, compared with 214.0 (IQR: 141.5–331.5), 193.5 (IQR: 114.2–344.5), and 144.0 (IQR: 75.2–205.0) cells/μL in the mild, moderate, and severe ARDS groups, respectively. A significant difference was observed across the four groups by Kruskal-Wallis test (H = 27.916, *p* < 0.001), and a significant decreasing trend was further confirmed by Jonckheere-Terpstra test for trend (Z = −5.415, *p* < 0.001) (Table 5).

**Table 5:** Comparison of CD3 + CD4 + T cell counts across ARDS severity groups.

ARDS severity	*n*	CD3 + CD4 + T cell count (cells/μL)	Statistical method	Test statistic	*p*-value
Overall	227	235.0 (143.0, 426.0)	Kruskal-Wallis test	H = 27.916	<0.001
Non-ARDS	100	378.0 (204.8, 533.8)			
Mild	55	214.0 (141.5, 331.5)			
Moderate	54	193.5 (114.2, 344.5)			
Severe	18	144.0 (75.2, 205.0)			
Trend analysis			Jonckheere-Terpstra test	Z = −5.415*	<0.001

Data are presented as median (interquartile range, Q1–Q3). The overall comparison among the four groups was performed using the Kruskal-Wallis test. The Jonckheere-Terpstra test was used for trend analysis across ARDS severity groups. * Standardized test statistic (negative value indicates a decreasing trend with increasing ARDS severity).

### 3.4 Subgroup analyses

**Figure 4:**
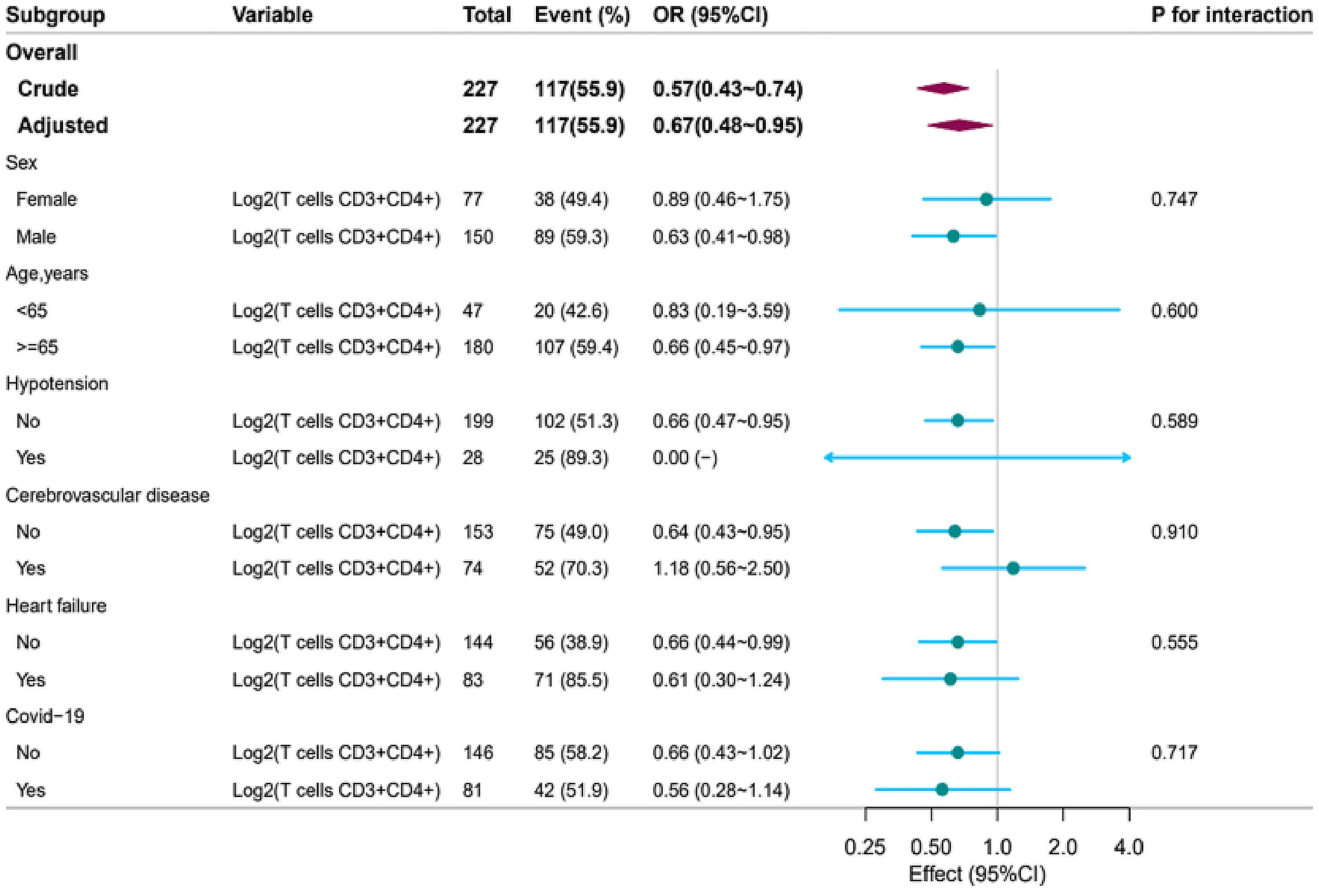
Subgroup analyses for the association of Log_2_ (T cells CD3^+^CD4^+^) and ARDS. OR, odds ratio; CI, confidence interval. Except for the stratification component itself, each stratification factor was adjusted for age, sex, albumin, Tbil, neutrophil count, C-reactive protein (CRP), procalcitonin (PCT), cerebrovascular disease, and heart failure.

Stratified analysis was conducted in several subgroups to evaluate potential effect modifications on the association between Log2 (T cells CD3 + CD4+) and ARDS. No significant interactions were observed in any subgroups after stratification by sex, age (<65 vs. ≥65 years), hypotension at admission, cerebrovascular disease, heart failure, and COVID-19 infection (all P for interaction >0.05) (Figure 4). Of note, the hypotension subgroup had a limited sample size, yielding an unstable confidence interval; thus, the result should be interpreted with caution.

## 4 Discussion

Pneumonia is a major global clinical and a leading cause of mortality worldwide (1). ARDS is a serious complication of pneumonia, and patients who develop ARDS have a significantly increased risk of death (3). The pathogenesis of ARDS is complex. The pathological core of ARDS has been considered to be diffuse alveolar damage and uncontrolled inflammatory response (15). However, growing evidence indicates that immune dysregulation-characterized by the coexistence of both excessive inflammation and immunosuppression-plays a pivotal role in the disease process (10). Recent studies have further underscored the heterogeneity of ARDS, revealing distinct clinical and biological phenotypes that are critical for advancing personalized treatment strategies (16). Among them, ARDS subtypes driven by pneumonia or sepsis are often accompanied by more pronounced immunosuppressive features. Especially in severe COVID-19, studies have clearly revealed that severe T cell exhaustion and dysfunction are one of the core mechanisms of disease severity (17). However, there is currently a lack of relevant studies in the general pneumonia population. Our study grouped general adult pneumonia patients based on the presence or absence of pneumonia-related ARDS and analyzed the differences in lymphocyte subset characteristics.

To improve the reliability of our findings, we used LASSO analysis to identify relevant variables, followed by logistic regression for further adjustment. The results showed that, after adjusting for confounding factors, Log2 (CD3^+^CD4^+^ T-cell count) remained independently associated with lower odds of ARDS (OR 0.67; 95% CI, 0.48–0.95; *p* = 0.023). Subgroup analysis further validated the robustness of these results. We explored the dose–response relationship between T cell (CD3 + CD4+) count and the presence of pneumonia-related ARDS using a restricted cubic spline model. The results revealed a significant non-linear association, exhibiting an inverse L-shaped relationship with a clear threshold effect. Specifically, a CD3 + CD4 + T cell count below approximately 227 cells/μL was associated with a markedly higher likelihood of ARDS, and this association strengthened as the count decreased further. This finding is consistent with the hypothesis that immune alterations are linked to ARDS pathogenesis. However, given the cross-sectional nature of our data, we are unable to determine the underlying mechanism responsible for this low count. It may reflect pre-existing immune compromise or acute stress-induced lymphopenia. Differentiating between these possibilities would require functional immune assessments and serial lymphocyte measurements. While our study cannot determine mechanism, prior research has explored potential causes for low CD3 + CD4 + T-cell counts in critical illness. For instance, a study by Mitsuyama et al. (18) suggests that under the stress of acute lung injury, factors such as cytokine storm and metabolic reprogramming may induce T cell apoptosis. Our preliminary study suggests that a low CD3 + CD4 + T-cell count is a common feature in adult pneumonia-related ARDS, a finding that warrants further investigation using appropriate functional and phenotypic assays. Consistent with a recent study by Yan et al. (19) demonstrating that decreased CD8^+^ T-cell counts were associated with ARDS development in septic patients, our findings also revealed significantly lower CD3^+^CD8^+^ T-cell counts in pneumonia patients with ARDS compared to those without. Although this difference was statistically significant in univariate analysis, it was not retained in the LASSO regression model for variable selection. This suggests that the independent predictive value of CD8^+^ T-cell counts may be attenuated when adjusted for other collinear factors, warranting further investigation in larger, prospective cohorts to clarify its potential role in ARDS pathogenesis.

This study is not without limitations. In interpreting our results, we must acknowledge the following limitations. First, despite our efforts to control for confounding factors, the single-center retrospective study design may mean that some unidentified variables could influence our findings. For example, although we excluded patients with long-term corticosteroid exposure and ensured that blood samples for lymphocyte subset analysis were collected within 24 h of admission and processed within 48 h to minimize the potential confounding effect of post-admission corticosteroid exposure on baseline lymphocyte measurements, we cannot completely rule out the possibility of short-term corticosteroid use prior to admission due to the retrospective nature of this study. Such pre-admission exposure could potentially influence lymphocyte counts and represents an inherent limitation of the study design. Second, ARDS status was determined at admission, while lymphocyte subsets were measured within 48 h of admission. We cannot establish whether reduced CD3^+^CD4^+^ T-cell counts preceded the development of ARDS or were a consequence of the acute inflammatory response associated with established ARDS or severe pneumonia. Consequently, our findings should be interpreted as demonstrating an association between CD3^+^CD4^+^ T-cell counts and ARDS, rather than a causal or protective effect. Future prospective cohort studies are warranted, incorporating functional immune assays to clarify the causal relationship between reduced CD3^+^CD4^+^ T-cell counts and the risk of developing ARDS. Concurrently, rigorous predictive performance analyses-including assessments of discrimination and calibration-should be conducted to validate whether this biomarker provides incremental value for early risk stratification beyond standard clinical variables. Third, potential selection bias may exist. During the study period, lymphocyte subset testing was not routinely performed for all pneumonia patients but was ordered by clinicians based on clinical judgment, typically for cases involving suspected immune dysfunction, severe infection, or admission to the ICU. Therefore, our study population may represent a subgroup of patients with more severe pneumonia, which limits the generalizability of our findings to the broader pneumonia population. Due to the unavailability of clinical data from untested patients, we were unable to directly compare the characteristics of included and excluded individuals. Future prospective studies with consecutive enrollment in unselected populations are warranted to validate our findings. Fourth, our study measured only absolute lymphocyte counts and did not include functional or phenotypic markers (e.g., activation or exhaustion markers). Therefore, while we observe an association between low CD3^+^CD4^+^ T-cell counts and ARDS, we cannot determine the underlying biological mechanism, such as whether this represents apoptosis, redistribution, or true immune exhaustion. Consequently, any interpretations regarding immune dysfunction remain speculative and should be viewed as hypothesis-generating. Fifth, the threshold analysis identifying approximately 227 cells/μL as a potential inflection point represents an exploratory finding derived from our dataset, rather than a definitive clinical cut-off. As with any data-driven threshold identification, this finding requires validation in independent external cohorts to confirm its stability and clinical applicability. Therefore, the precise numerical value of this threshold should be interpreted with caution until further validated in prospective studies.

## 5 Conclusion

Through the analysis of early lymphocyte subpopulations in patients with pneumonia, this study identifies an independent inverse association between reduced CD3^+^CD4^+^ T-cell counts and pneumonia-related ARDS. These findings suggest that quantitative lymphocyte abnormalities may contribute to the immunopathology of ARDS and extend observations from COVID-19 and sepsis to a broader pneumonia population. While the assessment of CD3^+^CD4^+^ T-cells may be valuable for identifying patients with higher odds of ARDS, our cross-sectional design prevent us from drawing conclusions about its clinical utility. Further research with functional immune assays and rigorous predictive performance analyses (including discrimination and calibration) in prospective cohorts is needed to determine whether this biomarker can meaningfully improve risk stratification and guide clinical management.

## Data Availability

The raw data supporting the conclusions of this article will be made available by the authors, without undue reservation.
